# Influence of Pretreatment Methods on Compressive Performance Improvement and Failure Mechanism Analysis of Recycled Aggregate Concrete

**DOI:** 10.3390/ma16103807

**Published:** 2023-05-18

**Authors:** Dongbin Lv, Kainan Huang, Wensheng Wang

**Affiliations:** 1 Guangxi Fuhe Expressway Co., Ltd., Hezhou 542800, China; 2 College of Transportation, Jilin University, Changchun 130025, China; 3 Guangxi Transportation Science and Technology Group Co., Ltd., Nanning 530007, China

**Keywords:** recycled aggregate, pretreatment method, compressive performance improvement, failure mechanism

## Abstract

The utilization of recycled aggregate can avert the squandering of resources and the destruction of the environment. Nevertheless, there exists a slew of old cement mortar and microcracks on the surface of recycled aggregate, which give rise to the poor performance of aggregates in concrete. In this study, for the sake of ameliorating this property of recycled aggregates, the surface of the recycled aggregates is covered with a layer of cement mortar to compensate for the microcracks on the surface and reinforce the bond between old cement mortar and aggregates. In order to demonstrate the influence of recycled aggregate by different cement mortar pretreatment methods, this study prepared natural aggregate concrete (NAC) and concretes with recycled aggregate after the wetting pretreatment (RAC-W) and cement mortar pretreatment (RAC-C), and conducted uniaxial compressive strength tests on different types of concrete at different curing ages. The test results indicated that the compressive strength of RAC-C at a 7 d curing age was higher than that of RAC-W and NAC, and the compressive strength of RAC-C at a 28 d curing age was higher than RAC-W but lower than NAC. The compressive strength of NAC and RAC-W at a 7 d curing age was about 70% of that at a 28 d curing age, and the compressive strength of RAC-C at a 7 d curing age was about 85–90% of that at a 28 d curing age. The compressive strength of RAC-C increased dramatically at the early stage, while the post-strength of the NAC and RAC-W groups increased rapidly. The fracture surface of RAC-W mainly occurred in the transition zone between the recycled aggregates and old cement mortar under the pressure of the uniaxial compressive load. However, the main failure of RAC-C was the crushing destruction of cement mortar. With changes in the amount of cement added beforehand, the proportion of aggregate damage and A-P interface damage of RAC-C also changed accordingly. Therefore, the recycled aggregate pretreated with cement mortar can significantly improve the compressive strength of recycled aggregate concrete. The optimal amount of pre-added cement was 25%, which is recommended for practical engineering.

## 1. Introduction

With the innovation of technology, plenty of concrete buildings need to be updated [1]. According to the relevant literature, about 40% of the construction garbage generated by the demolition of old buildings is concrete [2]. The recycling of concrete garbage not only satisfies the requirement of sustainable development, but also makes a huge contribution to the protection of the environment and the conservation of land resources [3]. At present, various types of recycled aggregates can replace natural aggregates to varying degrees for use in concrete materials, such as waste powder, fine and coarse marble aggregates [4,5], waste glass [6,7,8], recycled coal bottom ash [9], plastic waste [10], etc. With the increasing demand for sustainable development, recycled aggregate concrete (RAC), as a sustainable material, has received much attention in recent years due to its potential benefits in reducing environmental impact and conserving natural resources [11]. However, RAC has some drawbacks, such as low mechanical properties and poor durability than those of conventional concrete, which limit its widespread use in construction [12,13].

To address this issue, various pretreatment methods have been developed to enhance the mechanical properties of RAC [14,15,16,17]. These methods include surface modification, alkali activation, thermal treatment, and chemical treatment. Furthermore, the bond behaviour of passively confined RAC has been investigated based on centre pull-out tests [18]. Research about the preprocessing of recycled aggregate has already evolved. The effectiveness of these pretreatment methods on RAC has been studied by many researchers, and it has been found that they can significantly enhance the compressive strength and other mechanical properties of RAC [19,20,21]. Some researchers tried to remove the old cement mortar on the recycled aggregate with the preprocessing of HCI and Na_2_SO_4_ and found that the pretreatment of HCI can conspicuously enhance the compressive strength of RAC [22]. Furthermore, the silica fume solution was also used to improve the bond of the transition interface and fill the microcracks of recycled aggregate. Subsequently, the compactness and strength of the recycled aggregate were significantly improved [23]. In addition, removing the adhesion mortar was an effective method to improve the performance of recycled aggregates [15]. Meanwhile, there were also a few researchers who found that the concrete prepared by the pre-wetted recycled aggregate had the highest compressive strength [24] because the water plays an internal curing role in the concrete, which improved the compressive strength and freeze–thaw performance of the recycled concrete [25]. However, others found that recycled concrete prepared with dry aggregate had the best compressive strength; the concrete comprised of pre-wetted recycled aggregate had the best workability [26]. Through the uniaxial compression test of recycled aggregate concrete, researchers found that the compressive failure of recycled aggregate concrete mainly occurred in the interface transition zone of mortar and aggregate. Consequently, the performance of recycled aggregate concrete was significantly improved by increasing the strength of the interface transition zone [27]. Hou et al. found that the microcracks on the surface of the recycled aggregate would absorb cement particles. This process can enhance the transition zone between the new cement mortar and the old cement mortar, which results in a faster increase in the early compressive strength of the recycled concrete [28].

On the basis of previous research, the major approach to reusing concrete garbage is to convert it to recycled aggregate [24,29,30,31]. However, recycled aggregate is denser and different from natural aggregate [32]. Moreover, the surface of the recycled aggregate is covered with a layer of old cement mortar, and in the process of mechanical crushing, some microcracks are produced. These microcracks give rise to more water absorption and porosity. Subsequently, the workability of RAC is lower than that of natural aggregate concrete [33]. Hence, microcracks and old mortar on the surface of aggregate have a negative effect on the properties of recycled aggregate. Aggregate cracking notably reduces the performance of the entire structure, which is often investigated by numerical models in order to propose real strategies to prevent cracking phenomena. The discrete and smeared crack approaches provide reliable results in terms of crack patterns and tensile and compressive strength [34,35]. Hence, preprocessing the recycled aggregates would be a valid method to ameliorate the performance of the concrete. Some researchers introduced numerous methods to preprocess the aggregate, such as pre-wetting the aggregate and removing the old mortar adhered to the aggregate. Whereas, there remained relatively few applications of the method that blended the cement mortar and the aggregate preprocessed by cement paste directly. Furthermore, the paste wrapped on the aggregate would fill the cracks on the surface of the recycled aggregate and improve the bonding effect between the recycled aggregate and the old mortar, as well as the old mortar and the new mortar [36]. Compared with traditional methods, this way not only improves the performance of the concrete, but also saves time in the actual project.

This paper aims to investigate the influence of different pretreatment methods on the compressive performance improvement of RAC and analyze the failure mechanism of RAC under compression. The research will focus on two commonly used pretreatment methods, including wetting pretreatment and cement mortar pretreatment. Therefore, the paper’s innovation lies in exploring the effects of different treatments on recycled aggregate concrete’s properties and analyzing its failure mechanisms. The results of this study will provide valuable insights into the optimization of pretreatment methods for RAC, which can provide insights into the sustainable use of construction materials and lead to the development of more sustainable and high-performance construction materials.

## 2. Experimental Investigation

### 2.1. Raw Materials

**Figure 1:**
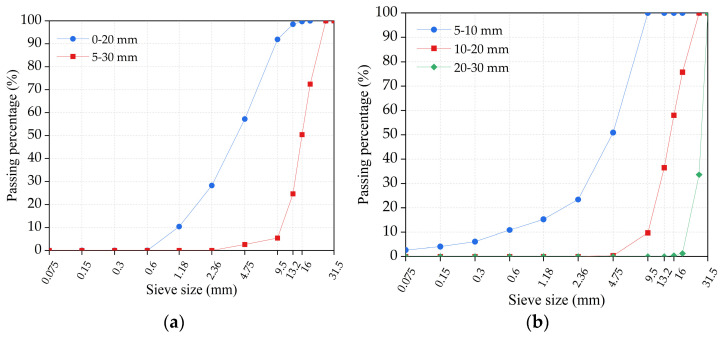
The sieve analysis results: (**a**) recycled coarse aggregate; (**b**) natural coarse aggregate.

In this study, the cement used in the experiment is ordinary Portland cement with a strength grade of 42.5 MPa, an initial setting time of 86 min and a final setting time of 142 min, which has 62.3% of CaO, 22.8% of SiO_2_, 5.6% of Al_2_O_3_, 4.4% of Fe_2_O_3_, 1.7% of MgO, etc. For the used coarse aggregates, the recycled aggregate was produced by crushing concrete waste from construction waste demolished in a project in Changchun, and the natural aggregate was basalt. Both recycled coarse aggregate and natural coarse aggregate were from Jilin Province, and the water absorption test results at different immersion times are summarized in Table 1. The crush index of recycled coarse aggregate and natural coarse aggregate was 10.96% and 4.08%, respectively. The sieve analysis results for both recycled coarse aggregate and natural coarse aggregate are shown in Figure 1. The fine aggregate used was natural sand, with an apparent density of 2541.0 kg/m^3^, a water absorption rate of 0.7%, and a fineness modulus of 2.38. Table 2 shows the particle size distribution of natural sand. In addition, the water used in the concrete was drinking water, and the additional agent was a polycarboxylic acid superplasticizer with a 25% water reduction rate.

### 2.2. Specimen Preparation and Experimental Method

In this study, two pretreatment methods were used to improve the performance of recycled aggregate, preparing the concrete specimens with recycled aggregate after the wetting pretreatment (i.e., RAC-W) and after the cement mortar pretreatment (i.e., RAC-C). At the same time, natural aggregate concrete (NAC) was also prepared as a control group for the following comparative analysis.

For the RAC-W specimen preparation, based on the water absorption test results of recycled coarse aggregate at different immersion times in Table 1, the recycled coarse aggregate immersed for 1 h was selected to carry out the wetting pretreatment, and then natural sand, cement, and water were poured into the mixer one by one. After mixing thoroughly, the RAC-W specimens were prepared.

The RAC-C specimen was divided into two groups, including the RAC-CA group and the RAC-CB group. Firstly, the recycled coarse aggregates with different particle sizes were poured into a mixer and mixed until the differently-sized recycled coarse aggregates were evenly distributed. Then, the cement and water were added to the mixer, with 20%, 25%, and 30% of their total amount, respectively. Next, the remaining cement, water, and natural sand were poured into the mixer to prepare cement mortar, mixing with the recycled aggregate after cement mortar pretreatment to prepare the RAC-CA specimens. On the other hand, the cement mortar pretreatment of recycled coarse aggregate for the RAC-CB group was the same as the RAC-CA group. Whereas the normal amount of cement, water, and natural sand—rather than the remaining or partial materials—were poured into the mixer to prepare the cement mortar. Then, the recycled coarse aggregates after cement mortar pretreatment were blended with the normal amount of cement mortar to prepare the RAC-CB specimens. The RAC-CB group used more cement and natural sand compared with the RAC-CA group; hence, the cost of the RAC-CB group increased by contrast with the RAC-CA group. The purpose of pouring the recycled aggregate after cement mortar pretreatment directly into the cement mortar is that the cement paste wrapped on the surface of the recycled aggregate could be prevented from washing away by adding water.

**Figure 2:**
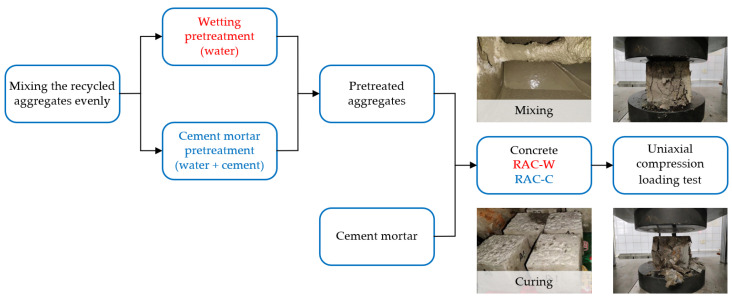
The flow diagram of RAC preparation and test.

The flow diagram of RAC-C preparation is shown in Figure 2. Based on the considerations, including the availability of materials, sustainability goals, and desired performance characteristics, the different types of concrete proportions are shown in Table 3. Specimens of 100 mm × 100 mm × 100 mm were produced and then placed in a standard curing room with a temperature of (20 ± 2) °C and humidity greater than 95% for curing. The uniaxial compression loading tests of different types of concrete (i.e., NAC, RAC-W, RAC-CA, and RAC-CB) were conducted using a mechanical testing machine, with a loading rate maintained at 0.05 MPa/s [37].

## 3. Results and Discussions

### 3.1. Analysis of Compressive Strength of Concrete at a 7-d Curing Age

**Figure 3:**
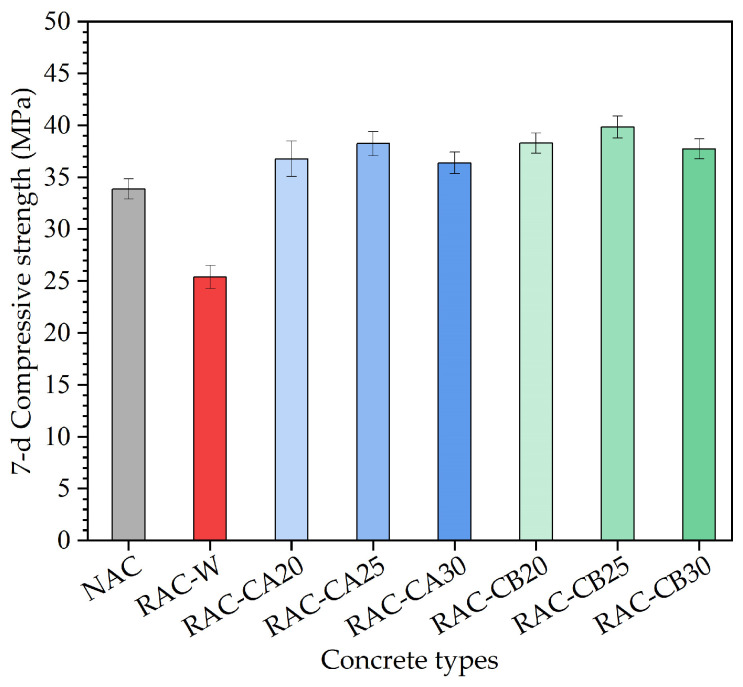
Bar chart of compressive strength for three types of concrete at a 7 d curing age.

In order to more intuitively display the changes in compressive strength of four different types of concrete, the bar charts of compressive strength results for these four types of concrete at a 7 d curing age are drawn in this study, as shown in Figure 3.

From the compressive strength results in Figure 3, it can be clearly seen that the compressive strengths of two RAC-C groups after two kinds of cement mortar pretreatment methods were generally higher than the NAC and RAC-W groups. For these two RAC-C groups, when the amount of cement and water added to the mixer in the pretreatment step was 20%, 25%, and 30%, respectively, the compressive strength of RAC-CA and RAC-CB showed a trend of first increasing and then decreasing. When the amount of pre-added cement and water reached 30%, after the 7 d curing age, the corresponding compressive strength of recycled aggregate concrete (i.e., RAC-CA30 and RAC-CB30) was the lowest among RAC-C20, RAC-C25, and RAC-C30. This may be because the cement mortar wrapped on the surface of recycled aggregate was thicker, and a higher cement content results in insufficient hydration reaction during a shorter curing age, resulting in a decrease in the compressive strength of concrete. When the amount of pre-added cement and water was 25%, the compressive strength of recycled aggregate concrete (i.e., RAC-CA25 and RAC-CB25) reached its maximum value in each RAC-C group. Therefore, based on the RAC-C compressive strength results at a 7-day curing age, it can be preliminarily predicted that the optimal amount of pre-added cement was 25%. Furthermore, it can be clearly seen that the compressive strength of the RAC-CB group was higher than that of the RAC-CA group as a whole. When the amount of cement added in the pretreatment step was 20% of the total, the compressive strength of the RAC-CB group (i.e., RAC-CB20) was about 4.1% higher than that of the RAC-CA20. For the 25% pre-added cement, the compressive strength of RAC-CB25 was about 4.2% higher than that of the RAC-CA25; for the 30% pre-added cement content, the compressive strength of the RAC-CB30 was about 3.8% higher than the RAC-CA30. In Table 3, the cement content of the RAC-CB group was higher than the RAC-CA group. Hence, the overall compressive strength of the RAC-CB group was higher than the RAC-CA group.

According to the compressive strength test results of the RAC-W and RAC-C groups, at a 7-day curing age, the compressive strength of the RAC-C group was higher than the RAC-W group. The average compressive strength for the 7-day curing RAC-CA group was about 46.0% higher than that of the RAC-W group, and the average compressive strength of the 7-day curing age RAC-CB group was about 51.9% higher than that of the RAC-W group. The reason may be that part of the water for the wetting pretreatment would become the water for mixing [38], which resulted in an increase in the water–cement ratio, thus reducing the compressive strength. In addition, part of the water absorbed by the recycled aggregate in the wetting pretreatment step would return to the cement mortar ascribed to the hydrostatic pressure during the process of mixing [39]. The water–cement ratio of concrete would also be increased, and therefore, the compressive strength was reduced. Moreover, the recycled aggregate of the RAC-CA and RAC-CB groups is pretreated with cement mortar, and thus the microcracks on the surface of the recycled aggregate would be filled by the cement mortar. The cement mortar pretreatment would strengthen the combination of the old cement mortar and the recycled aggregate and improve the integrity of the recycled aggregate, sequentially increasing the compressive strength of the recycled aggregate concrete.

At a 7-day curing age, the compressive strength of the RAC-C group was higher than that of NAC based on the comparison of the experimental results. The average compressive strength at a 7-day curing age of the RAC-CA and RAC-CB groups was 9.8% and 14.3% higher than that of the NAC group, respectively. The reasons are that the overall performances of the recycled aggregate of the RAC-CA and RAC-CB groups were improved with the effect of pre-added cement mortar, and part of the water for mixing was still absorbed by the recycled aggregate during the blending process. However, the recycled aggregate is pretreated with cement mortar, which would result in a decrease in the water–cement ratio. Because the initial strength of concrete is mainly determined by the water–cement ratio, the compressive strength of the 7-day curing age RAC-CA and RAC-CB groups was higher than that of the NAC group.

### 3.2. Analysis of Compressive Strength of Concrete at a 28 d Curing Age

**Figure 4:**
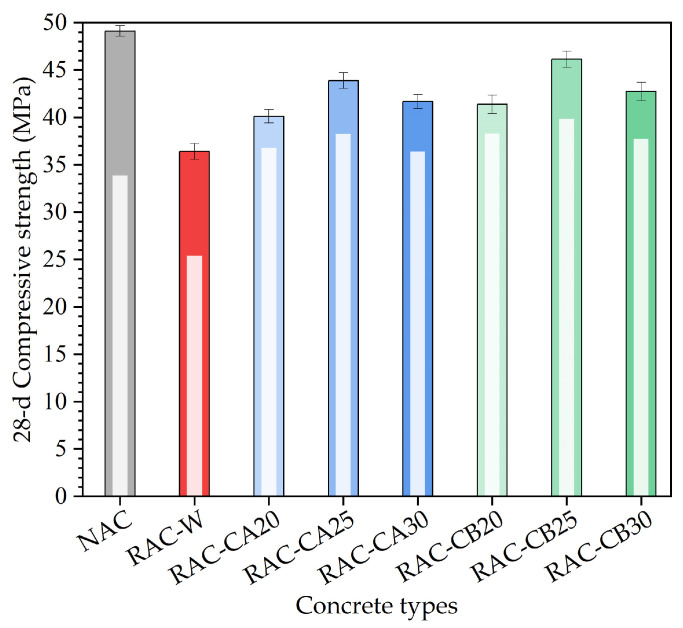
Bar chart of compressive strength for three types of concrete at a 28 d curing age.

Similarly, in order to clearly display the changes in compressive strength of different types of concrete at a 28 d curing age, a bar chart of compressive strength results for these four types of concrete is shown in Figure 4.

According to the compressive strength results in Figure 4, compared to the compressive strength at a 7 d curing age (shown in Figure 3), the compressive strength of different types of concrete at a 28 d curing age was improved to varying degrees. The compressive strength of the 28 d curing age RAC-CB group was higher than that of the RAC-CA group in general. When the amount of cement added in the pretreatment step reached 20%, 25%, and 30%, the compressive strength of the RAC-CB group was 3.2%, 4.9%, and 2.6% higher than that of the RAC-CA group, respectively. This is because the sand ratio of the RAC-CB group was lower, which gives rise to the stronger adhesion of cement mortar and recycled aggregate. Thus, the strength of the RAC-CB group was higher than that of the RAC-CA group. In addition, the compressive strength gap between the RAC-CA and RAC-CB groups decreased, compared with the compressive strength of the RAC-C type at a 7 d curing age. The reason might be that the cement amount of the RAC-CB group was relatively larger, which resulted in a relative weakness of the skeleton effect of the aggregate [40]. Consequently, the compressive strength gap between these two groups of RAC-CA and RAC-CB was relatively reduced. When the amount of pre-added cement was 30%, the compressive strength at a 28 d curing age of these two groups had a significant increase compared with the 7 d curing age. The reason is that the cement mortar on the surface of the recycled aggregate was cured for a longer time, which has already formed the hard cement stone giving rise to greater strength. When the amount of pre-added cement was 25%, the compressive strength of concrete at a 28 d curing age for RAC-C was the largest. Taking into account the compressive strengths at the 7 d and 28 d curing ages, it can be inferred that the optimal amount of pre-added cement is 25%.

In Figure 4, it can be seen from the compressive test results that the compressive strength at a 28 d curing age of the RAC-CA and RAC-CB groups is 44.8% and 57.4%, respectively, higher than that of the RAC-W group on average. The reason is that the microcracks of the recycled aggregate in the RAC-C group have been filled with cement mortar, and the surface of the recycled aggregate is covered with a layer of hard cement, which improves the performance of the recycled aggregate. Therefore, the compressive strength of the RAC-C group is higher than that of the RAC-W group. The average compressive strength at a 28 d curing age of the RAC-CA and RAC-CB groups is 14.5% and 11.4% lower than that of the NAC group, respectively. The reason may be that the compressive strength at the later period of concrete mainly depends on the quality of the aggregate. Although the quality of the recycled aggregate in the pretreatment step has been improved with cement mortar, its mechanical properties are still inferior compared with the natural aggregate. Thus, the compressive strength of the RAC-CA and RAC-CB groups was lower than that of the NAC group at the later period.

**Figure 5:**
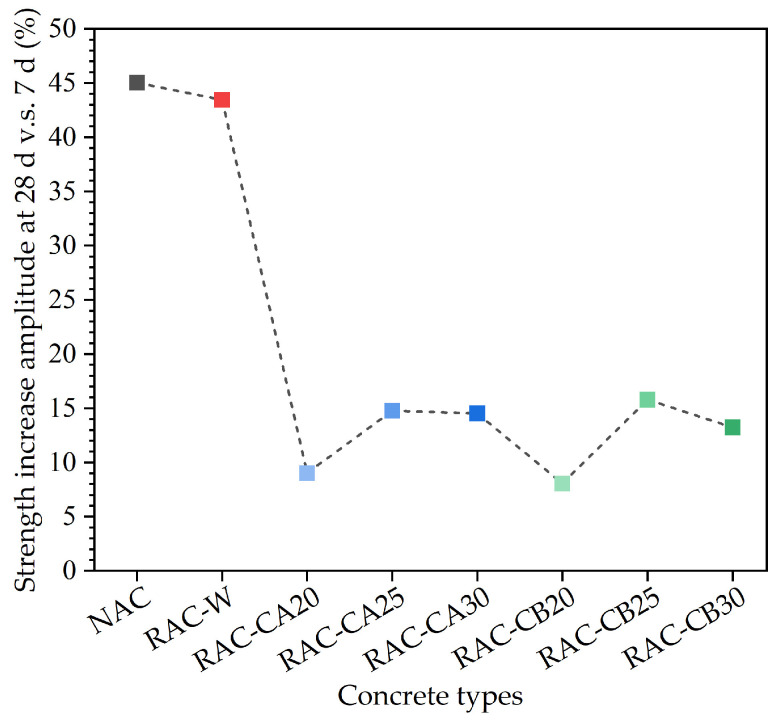
The compressive strength amplitude increase at the curing age of 28 d vs. 7 d.

The compressive strength ratio of different concrete types at the 7 d curing age to 28 d curing age was calculated and is listed in Table 4. From the compressive strength ratio results shown in Table 4, it can be seen that the compressive strength of the NAC and RAC-W groups at a 7 d curing age was about 70% of the compressive strength at a 28 d curing age. The compressive strength of the RAC-CA and RAC-CB groups at a 7 d curing age was about 85–90% of the compressive strength at a 28 d curing age. Figure 5 shows the compressive strength increases amplitude at the curing age of 28 d vs. 7 d. This shows that the posted strength of the NAC and RAC-W groups increases rapidly; however, the compressive strength amplitude increase of the RAC-CA and RAC-CB groups at a 28 d curing age was relatively smaller. Among the RAC-C group, 25% of the pre-added cement would lead to the obvious compressive strength increase at a 28 d curing age.

### 3.3. Failure Mechanism of Recycled Aggregate Concrete under the Compressive Effect

The fracture of the NAC group under the pressure of a uniaxial compressive load would be mainly located in the transition zone between the aggregates and cement mortar. This is because aggregate and cement mortar are considered two kinds of media. When different kinds of media are blended together, their connection interface is considered to be the weakest position in the mixture of the two media. As a result, when the NAC group was damaged under pressure, the fracture mostly appeared at the connection between aggregates and cement mortar.

**Figure 6:**
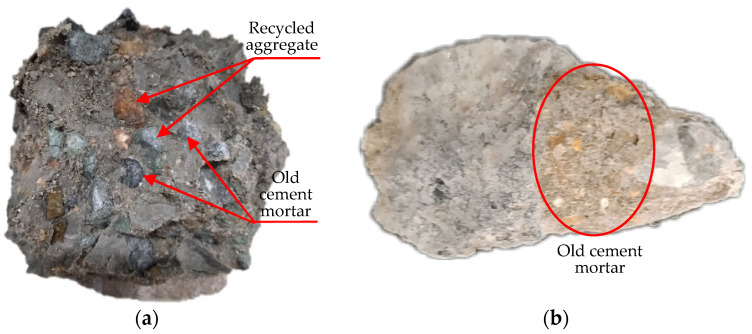
The fracture of the RAC-W group under the pressure of uniaxial compressive load: (**a**) broken diagram of RAC-W; (**b**) surface of recycled aggregate.

For the RAC-W group, the compressive test results show that the fracture surface mainly occurs in the transition zone between the recycled aggregates and old cement mortar under the pressure of the uniaxial compressive load, as shown in Figure 6a. The recycled aggregate in Figure 6b is usually attached to a layer of old cement mortar on the surface. Once it is used to form the concrete specimen, the recycled aggregates and old cement mortar would be wrapped in new cement mortar. In general, on account of mechanical crushing, the old cement mortar of the recycled aggregates on the surface gets rougher, which contributes to a close combination between the new cement mortar and the old cement mortar. However, the old cement mortar has a lower performance attribute to ageing and vibration intensity during the mechanical crushing process. Hence, the strength of the transition zone between the old mortar and the new mortar is greater than the strength of the transition zone between the recycled aggregates and the old mortar, which would lead to the recycled aggregates being mostly exposed on the surface when the RAC-W group is damaged under pressure.

**Figure 7:**
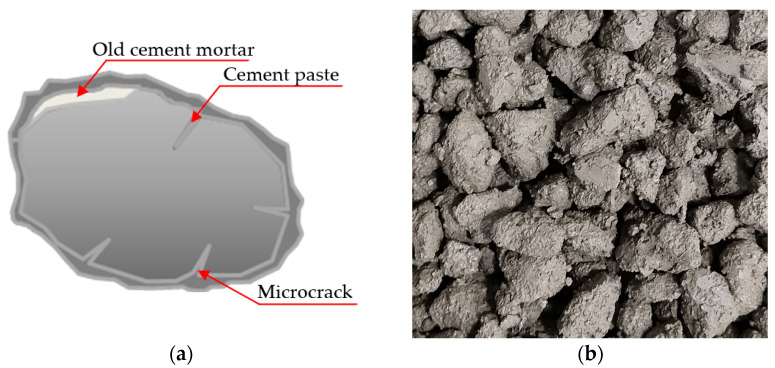
The RAC-C group after the cement mortar pretreatment: (**a**) structure of the recycled aggregate; (**b**) the real pretreated aggregates.

From the above analysis, it can be seen that the transition zone between the recycled aggregates and old cement mortar is the weakest position of the recycled aggregate concrete. Consequently, it would be a valid way to strengthen the mechanical properties of recycled concrete by strengthening the performance of the transition zone. In this study, the surface of the recycled aggregates is covered with a layer of cement mortar, which can form the shell structure. The structure of the recycled aggregate after the cement mortar pretreatment and the real pretreated aggregate are shown in Figure 7, in which the microcracks on the surface of the recycled aggregate are fully filled by the cement mortar. Part of the cement mortar is absorbed by the old cement mortar on the surface of the aggregate, which improves the properties of the old cement mortar. Moreover, the cement mortar would also strengthen the bond between the recycled aggregate and the old cement mortar. Therefore, the recycled aggregates pretreated with cement mortar have an overall increase in positive properties. Meanwhile, it can be found through the compression test that the main failure of the RAC-C group is the crushing failure of cement mortar, accompanied by different amounts of destructed aggregates and the aggregate–cement paste interface (i.e., A-P interface). The failure mainly occurs at the transition zone between old cement mortar and cement paste and the transition zone between aggregate and cement paste.

In this study, based on the software ImagePro Plus 6.0, the ratio of aggregate damage and A-P interface damage in the RAC-C group could be calculated. To distinguish the aggregate damage and A-P interface damage from the broken RAC specimens, ImagePro Plus software was used for image analysis as follows: First, the acquired RAC cross-sectional image was imported into the ImagePro Plus software. Then, the built-in image processing functions of the software were used, such as binarization and edge detection, to extract the damaged area of the aggregate damage and A-P interface damage. Next, using the software’s area statistics function, the area of the aggregate damage and A-P interface damage within the entire statistical area could be calculated. Finally, the ratio of the area of the aggregate damage and A-P interface damage could be used to calculate their respective proportions in the entire statistical area. The area ratio results of aggregate damage and A-P interface damage in the RAC-C group are listed as shown in Table 5. From the results, we see that as the proportion of aggregate damage increases, the compressive strength of the RAC-C group has a growing tendency and as the amount of pre-added cement increases, the proportion of A-P interface damage decreases.

**Figure 8:**
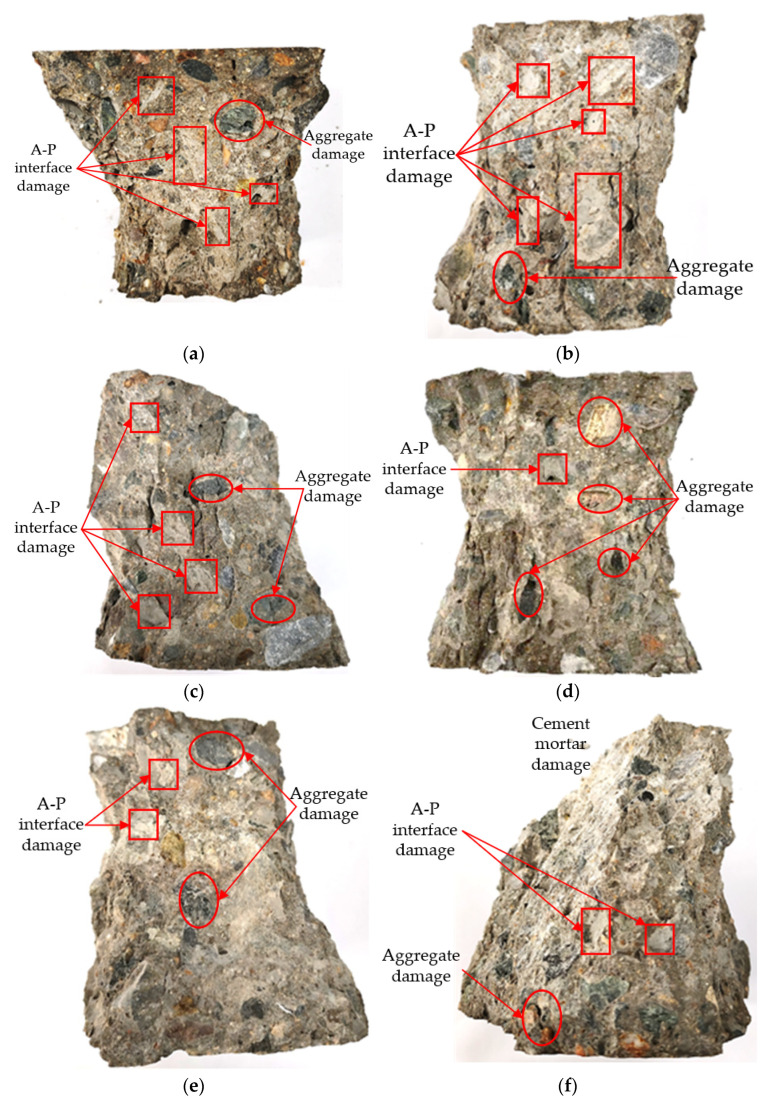
The damage comparison for the aggregate and A-P interface in the RAC-C group: (**a**) RAC-CA20; (**b**) RAC-CB20; (**c**) RAC-CA25; (**d**) RAC-CB25; (**e**) RAC-CA30; (**f**) RAC-CB30.

The comparison of aggregate and A-P interface damages in the RAC-CA and RAC-CB groups is shown in Figure 7. According to Table 5, the concrete of the RAC-CA20 group had relatively more damage to the A-P interface compared with the aggregate damage. The reasons are that the RAC-CA20 group has a small amount of pre-added cement, and the shell structure formed on the surface of the aggregate is relatively thin, which results in the inferior strength of the shell structure. Thus, the fracture of the RAC-CA20 concrete mainly appeared at the A-P interface, as shown in Figure 8a. The failure mode of the RAC-CB group was similar to that of the RAC-CA group; because there was comparatively less cement added in the pretreatment step, the shell structure formed on the surface of the aggregates was thinner, so the strength of the RAC-CA group was insufficient, easily resulting in damage, as shown in Figure 8b.

From Table 5, the ratio of aggregate damage in the RAC-CA25 group relatively increases, which also has more damage at the A-P interface. This is ascribed to the relatively smaller amount of cement mortar in the RAC-CA25 group compared with the RAC-CA20 group, and hence the main strength of concrete in this group is served by the embedding effect between the aggregates. As a result, during the compression test, most of the compressive load is resisted by the aggregates, which gives rise to a partial crushing failure of aggregates. Once the aggregate is broken, the A-P interface would also be destroyed, as shown in Figure 8c. Compared with the damage of the A-P interface, there were more crushing failures of the aggregate that occurred in the RAC-CB25 group. The reasons are that the quantity of the cement mortar was larger compared to the RAC-CA25 group, which contributes to better adhesion to the aggregates, enhancing their embedding effect. Consequently, when a small portion of cement mortar and the A-P interface are destroyed under the compressive load, the aggregate becomes the main bearer of the load, which eventually gives rise to the crushing failure of the aggregate, as shown in Figure 8d.

By comparing the RAC-CA30 and RAC-CB30 groups, relatively less damage of the A-P interface and aggregate occurred in the RAC-CA30 group. The crushing failure of cement mortar is the main failure form. The reasons are that the RAC-CA30 group has a greater amount of cement added in the pretreatment step, and hence the shell structure is continuous and complete and formed on the surface of the aggregates. In addition, the sand ratio of the RAC-CA30 group was larger than the RAC-CB30 group, which resulted in the relatively lower strength of the cement mortar. Consequently, almost all of the destruction of the RAC-CA30 group was caused by the crushing failure of the cement mortar. There were fewer exposed aggregates on the surface after the compressive failure, as shown in Figure 8e. The dominant destruction of the RAC-CB30 group was the crushing failure of cement mortar with little damage to the A-P interface and aggregates. This is because there was an enormous amount of cement added in the pretreatment step of the RAC-CB30 group. Thus, it is thicker for the shell structure wrapped on the surface of the aggregate. In addition, the amount of cement mortar was huge, which contributed to a relatively weakened embedding effect of the aggregate. Therefore, when the concrete was damaged under compression, the cement mortar bore most of the load, so the RAC-CB30 group was mainly damaged by cement mortar, as shown in Figure 8f.

## 4. Conclusions

This study prepared natural aggregate concrete (NAC) and concretes with recycled aggregate after the wetting pretreatment (RAC-W) and after the cement mortar pretreatment (RAC-C), and conducted uniaxial compressive strength tests on different types of concrete at different curing ages. Based on the compression test results and failure interfaces, the failure mechanisms of different types of concrete were analyzed. The following conclusions are drawn:

(1) The compressive strength of the RAC-C group, including RAC-CA and RAC-CB, after two kinds of cement mortar pretreatment methods at a 7 d curing age is higher than that of RAC-W and NAC, and the compressive strength of the RAC-C group at a 28 d curing age is higher than RAC-W but lower than NAC. Therefore, it may be a suitable option for projects that require quick strength development.
(2) At a 7 d curing age, the compressive strength of RAC-CB is about 3.8–4.2% higher than that of the RAC-CA, the compressive strength of RAC-CA is about 46.0–51.9% higher than that of RAC-W, the compressive strength of RAC-C is about 9.8–14.3% higher than that of NAC.
(3) At a 28 d curing age, the compressive strength of RAC-CB is about 2.6–4.9% higher than that of the RAC-CA, the compressive strength of RAC-CA is about 44.8–57.4% higher than that of RAC-W, the compressive strength of RAC-C is about 11.4–14.5% lower than that of NAC.
(4) The compressive strength of NAC and RAC-W at a 7 d curing age is about 70% of that at a 28 d curing age, and the compressive strength of RAC-C at a 7 d curing age is about 85–90% of that at a 28 d curing age. The compressive strength of RAC-C increased dramatically at the early stage, while the post-strength of the NAC and RAC-W groups increased rapidly.
(5) The fracture surface of RAC-W mainly occurs in the transition zone between the recycled aggregates and old cement mortar under the pressure of the uniaxial compressive load. The main failure of RAC-C is the crushing destruction of cement mortar. With the amount of cement added beforehand changed, the proportion of aggregate damage and A-P interface damage of RAC-C also changed accordingly.

Therefore, the appropriate type of concrete should be selected based on the project’s specific requirements. Concretes with recycled aggregate after the cement mortar pretreatment are suggested to improve the utilization rate of recycled aggregate and the compressive strength of concrete. The curing age and failure mechanisms should be considered while selecting the appropriate type of concrete.

## Figures and Tables

**Table 1:** Water absorption test results of recycled coarse aggregate at different immersion times.

Immersion Time (h)	1/6	1/3	0.5	1	2	3
Water absorption (%)	6.52	6.58	6.77	6.82	6.85	7.03

**Table 2:** Particle size distribution of natural sand.

Mesh Size (mm)	4.75	2.36	1.18	0.6	0.3	0.15
Cumulative residual percentage (%)	5.1	19.3	27.9	4.26	81.8	92.5

**Table 3:** The proportion of different types of concrete.

Type	Superplasticizer (%)	Ingredients (kg/m^3^)					
		Recycled Coarse Aggregate	Natural Coarse Aggregate	Total Cement	Total Water	Pre-Add Cement	Pre-Add Water
NAC	0.5	0	1888.4	400	160	0	0
RAC-W	0.5	1191.9	0	400	160	0	0
RAC-CA20	1	1191.9	0	400	160	80	32
RAC-CA25	1	1191.9	0	400	160	100	40
RAC-CA30	1	1191.9	0	400	160	120	48
RAC-CB20	1	1191.9	0	480	192	80	32
RAC-CB25	1	1191.9	0	500	200	100	40
RAC-CB30	1	1191.9	0	520	208	120	48

**Table 4:** The compressive strength ratio of 7 d curing age concrete to 28 d curing age concrete.

Type	NAC	RAC-W	RAC-CA20	RAC-CA25	RAC-CA30	RAC-CB20	RAC-CB25	RAC-CB30
Ratio (%)	68.9	69.7	91.6	86.8	87.3	92.3	86.2	88.3

**Table 5:** The ratio of aggregate damage and A-P interface damage in the RAC-C group.

Damage	RAC-CA20	RAC-CB20	RAC-CA25	RAC-CB25	RAC-CA30	RAC-CB30
Aggregate damage (%)	1.01	1.18	3.32	3.95	1.36	1.42
A-P interface damage (%)	6.37	8.15	5.84	2.91	3.04	1.72

## Data Availability

Not applicable.
